# Genome-Wide Mutation Avalanches Induced in Diploid Yeast Cells by a Base Analog or an APOBEC Deaminase

**DOI:** 10.1371/journal.pgen.1003736

**Published:** 2013-09-05

**Authors:** Artem G. Lada, Elena I. Stepchenkova, Irina S. R. Waisertreiger, Vladimir N. Noskov, Alok Dhar, James D. Eudy, Robert J. Boissy, Masayuki Hirano, Igor B. Rogozin, Youri I. Pavlov

**Affiliations:** 1 Eppley Institute for Research in Cancer and Allied Diseases, University of Nebraska Medical Center, Omaha, Nebraska, United States of America; 2 Saint Petersburg Branch of Vavilov Institute of General Genetics, St. Petersburg, Russia; 3 Department of Genetics, Saint Petersburg University, St. Petersburg, Russia; 4 J. Craig Venter Institute, Rockville, Maryland, United States of America; 5 Department of Genetics, Cell Biology and Anatomy and Munroe-Meyer Institute, University of Nebraska Medical Center, Omaha, Nebraska, United States of America; 6 Department of Internal Medicine, University of Nebraska Medical Center, Omaha, Nebraska, United States of America; 7 Emory Vaccine Center, Department of Pathology and Laboratory Medicine, Emory University, Atlanta, Georgia, United States of America; 8 National Center for Biotechnology Information, National Library of Medicine, National Institutes of Health, Bethesda, Maryland, United States of America; University of Washington, United States of America

## Abstract

Genetic information should be accurately transmitted from cell to cell; conversely, the adaptation in evolution and disease is fueled by mutations. In the case of cancer development, multiple genetic changes happen in somatic diploid cells. Most classic studies of the molecular mechanisms of mutagenesis have been performed in haploids. We demonstrate that the parameters of the mutation process are different in diploid cell populations. The genomes of drug-resistant mutants induced in yeast diploids by base analog 6-hydroxylaminopurine (HAP) or AID/APOBEC cytosine deaminase PmCDA1 from lamprey carried a stunning load of thousands of unselected mutations. Haploid mutants contained almost an order of magnitude fewer mutations. To explain this, we propose that the distribution of induced mutation rates in the cell population is uneven. The mutants in diploids with coincidental mutations in the two copies of the reporter gene arise from a fraction of cells that are transiently hypersensitive to the mutagenic action of a given mutagen. The progeny of such cells were never recovered in haploids due to the lethality caused by the inactivation of single-copy essential genes in cells with too many induced mutations. In diploid cells, the progeny of hypersensitive cells survived, but their genomes were saturated by heterozygous mutations. The reason for the hypermutability of cells could be transient faults of the mutation prevention pathways, like sanitization of nucleotide pools for HAP or an elevated expression of the *PmCDA1* gene or the temporary inability of the destruction of the deaminase. The hypothesis on spikes of mutability may explain the sudden acquisition of multiple mutational changes during evolution and carcinogenesis.

## Author Summary

Evolution and carcinogenesis are driven by mutations. Cells maintain constant mutation rates and can afford only transient mutagenesis bursts for adaptation. The nature of the mutational avalanches is not very clear. We sequenced the whole genomes of mutants induced in haploid and diploid yeast by nucleobase analog HAP and by DNA editing cytosine deaminase. Mutants selected in diploids are saturated with passenger mutations. Far fewer mutations are found in haploid mutants. Treatment with a mutagen without selection results in intermediate mutagenesis. The observed transient hypermutability of diploids under mutagenic insult helps to explain the wellspring of mutations that arise during evolution and carcinogenesis.

## Introduction

The precise balance between genome stability and mutagenesis is vital for the survival of a species [1], [2], [3]. It ensures the maintenance of the optimal combinations and frequencies of alleles with high fitness and, simultaneously, the introduction of new mutations that are the raw material for the natural selection that drives adaptation in a changing environment. A wealth of data indicate that this balance shifts toward higher mutation rates during sub-optimal conditions, and then returns to normal levels ([2], [4], [5] and references therein). Similar mechanisms have been proposed to explain the evolution of tumors [6], [7]. Sequencing of cancer genomes shows that tumor genomes are highly enriched with mutations [8], [9]. The accumulated mutation load cannot be explained by normal mutation rates and requires highly mutable cells ([10], [11]; reviewed in [12]). A stable mutator phenotype would inexorably reduce tumor fitness due to the accumulation of mutations in regulatory and essential genes. In order to account for this discrepancy, it has been hypothesized that the mutator phenotype in cancer is transient [13], [14]. Spikes of hypermutability can be caused by epigenetic changes and/or the defective regulation of DNA repair and replication [6], abnormally high expression of DNA editing deaminases [15], [16] and other processes.

**Figure 1:**
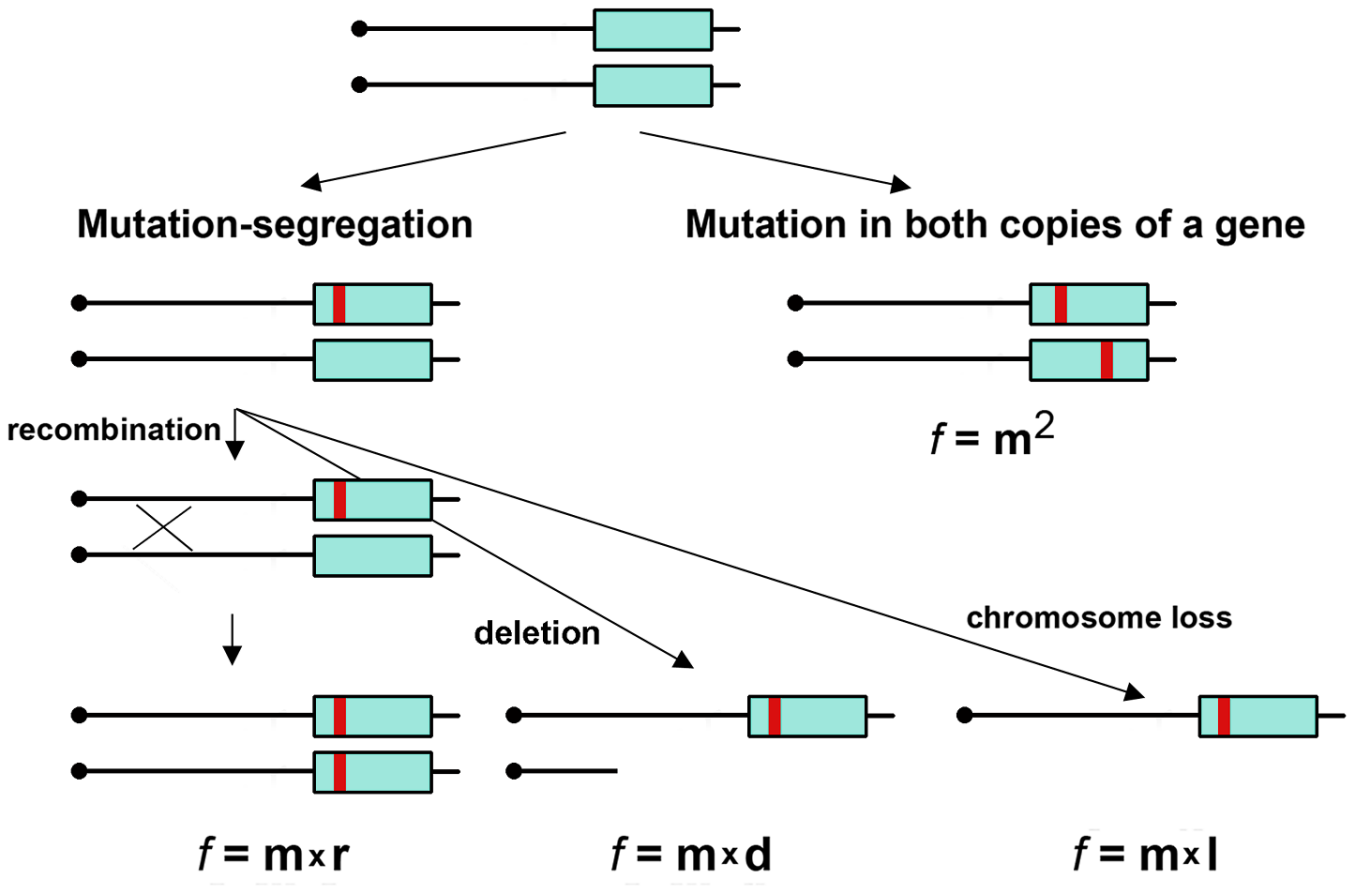
Two mechanisms of generation of mutants in diploids. Mutation (red bar) in a gene (blue rectangle) occurring with frequency “m” will lead to a phenotypic change in diploid, either by a concomitant loss of heterozygosity (by recombination with frequency “r”) or deletion (“d”) or chromosome information loss/inactivation (“l”). The frequency of such events “f” should be the product of the frequencies of each independent event. Mutations can also occur independently in both alleles of a diploid but at different sites, and their frequency should be the square of “m”.

Another layer of complexity is added by the fact that the mechanisms of the appearance of mutants are different in haploid and diploid organisms. In haploid cells, a mutation-causing defect of the gene product is expressed immediately. In diploid cells, a wild-type allele will mask a recessive mutation, and only the effects of dominant mutations will be observed (Fig. 1). For recessive mutations, the mutant phenotype will only be expressed in diploid cells when the second allele is inactivated. This can occur in various ways. First, either gene conversion or recombination between the mutated allele and the centromere will lead to a reduction to homozygosity. Second, chromosome loss or deletion of the region encoding the wild-type allele will result in a reduction to hemizygosity. Third, the wild-type allele may acquire an independent, typically heteroallelic mutation. The classic example illustrating the importance of two-step mutagenesis is Knudson's theory of retinoblastoma development via the inactivation of both alleles of a tumor suppressor gene [17], [18].

If measured by phenotypic change, mutation frequency should be much lower in diploids than haploids (Fig. 1); however, in yeast, it is only several-fold less ([19] and references therein). Most mutagens act in yeast by a two-step mechanism involving mutation and segregation, because they induce a high frequency of recombination events [20], while replication infidelity caused by non-recombinogenic base analogs or proofreading exonuclease defects somehow induces a high level of independent mutations in both homologs [21], [22].

Most of our knowledge of the mechanisms of mutagenesis comes from classical studies in haploid models, such as *E. coli*, haploid yeast strains, or *Drosophila* germ cells. The molecular mechanisms of mutagenesis in diploid cells have not been studied in-depth. In this work, we induced mutations in isogenic haploid and diploid yeast using one of two different types of mutagens that generate non-canonical bases in DNA: the base analog 6-hydroxylaminopurine (HAP), and ectopically produced editing cytosine deaminase PmCDA1 from sea lamprey. We have chosen these mutagens and genetic backgrounds to avoid an induction of recombination by mutagens. Yeast is characterized by high recombination. Our conditions were well-suited for study of mutagenesis more closely resembling the processes in human cells, when recombination is rare.

HAP and PmCDA1 enhance replication infidelity and create a mutator phenotype on demand. HAP is incorporated during the growth in a media with analog and rapidly wiped out from cells after transfer to the medium without it. It is known that nucleotide pools are constantly and rapidly renewed in yeast cells [23]. The expression of *PmCDA1* in our system is under the control of a regulatable promoter and could be turned on and off. After mutagenic treatment we selected forward mutants resistant to antibiotic canavanine or toxic drug 5-fluoroorotic acid (5-FOA) and resequenced their genomes. This allowed for the determination of accumulated DNA sequence changes specific for each mutagen. The numbers of induced base substitutions were more than an order of magnitude higher in diploid mutants than in haploid mutants. The genomes of diploid clones treated with either mutagen but not selected for resistance also contained significantly less mutations than the diploid mutant clones. This indicates the heterogeneity in mutability between different cells and proves that selected mutants came from a fraction of cells that experienced the most dramatic mutagenesis. We call such cells hypermutable. Diploid hypermutated cells survived, because most of the induced mutations were recessive and did not result in phenotypic changes when heterozygous. Haploids with similar levels of mutagenesis die due to inactivation of essential genes. For the first time, to our knowledge, this work suggests that cells have a wide range of mutability in a genetically homogenous population of eukaryotic cells exposed to a mutagen. This may explain the rapid appearance of mutations (mutation avalanches and recently discovered kataegis) in evolution and disease progression, especially in sporadic cancer.

## Results

### HAP and PmCDA1 are highly mutagenic in haploid and diploid yeast

**Figure 2:**
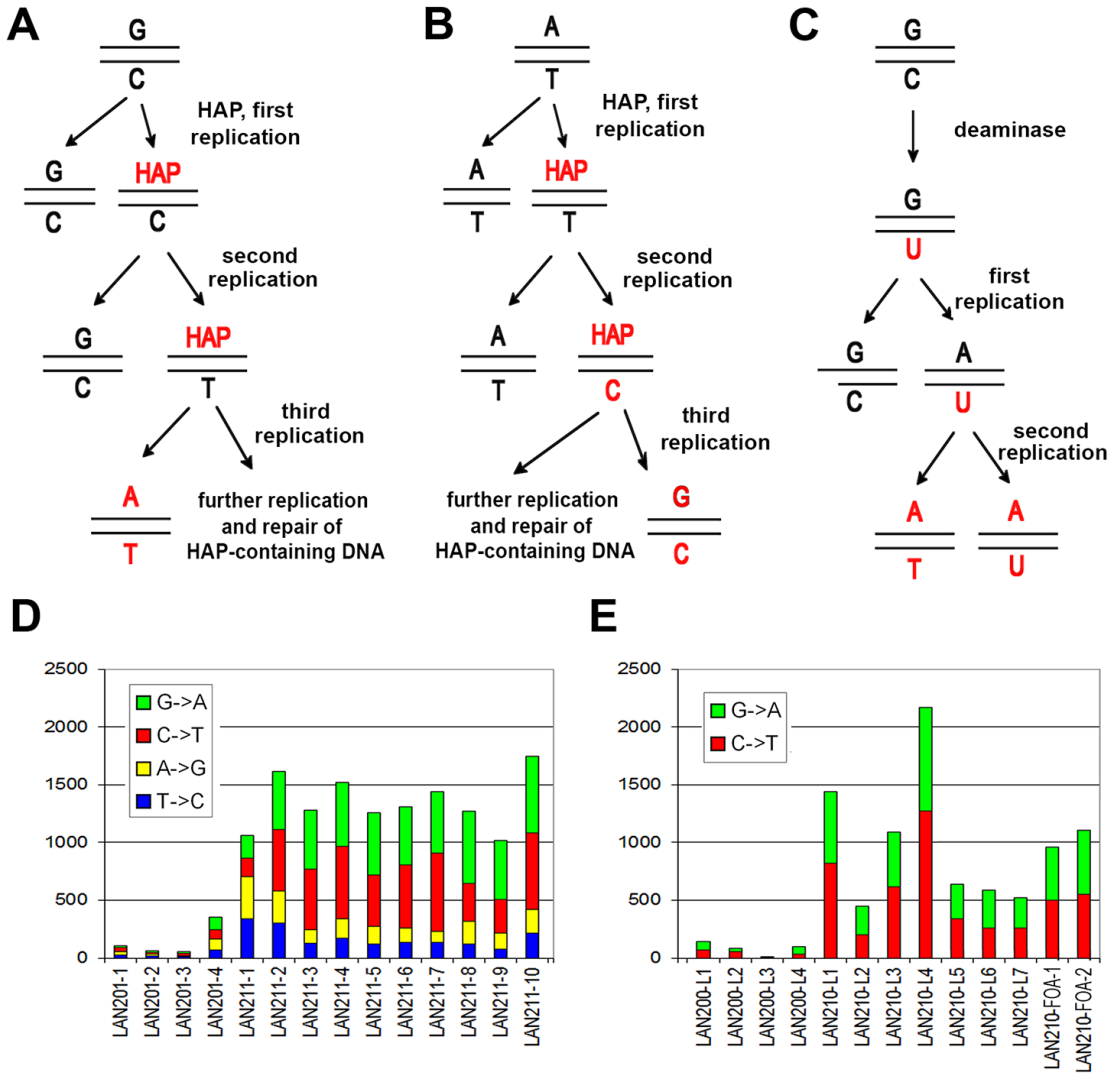
Induction of mutations by HAP and PmCDA1. **A.** Induction of G-C to A-T transitions by the mechanism of HAP misincorporation. **B.** Induction of A-T to G-C transition mutations by the mechanism of misincorporation opposite HAP. **C.** Induction of G-C to A-T mutations by cytosine deamination followed by incorporation of adenine opposite uracil. **D.** Numbers of SNVs induced by HAP in haploid (LAN201-1 - LAN201-4) and diploid (LAN211-1 – LAN211-10) strains. The proportions of substitution types are shown by color. **E.** Numbers of SNVs induced in haploid (LAN200-L1 - LAN200-L4) and diploid (LAN210-L1 – LAN210-L7), PmCDA1-induced mutants CAN^R^ mutants, and diploid PmCDA1-induced FOA^R^ mutants (LAN210-FOA-1 and LAN210-FOA-2). Proportions of substitution types are shown by color.

HAP is an adenine base analog that has an ambiguous base-pairing capacity. In imine form it can pair with thymine, whereas in its rarer amine form it pairs with cytosine. HAP is a universal mutagen that is active in most organisms, from humans to bacteria and their phages [24], [25]. The conversion of HAP in cells to the corresponding deoxyribonucleotide triphosphate (dHAPTP), followed by its incorporation into DNA by replicative polymerases, results in A-T to G-C and G-C to A-T transition mutations (see Fig. 2A, 2B) [26], [27], [28], [29], [30]. PmCDA1 belongs to the AID/APOBEC superfamily of editing deaminases [31], [32]. These enzymes are found in different vertebrate species and perform a variety of functions, including immunoglobulin gene diversification (AID), RNA editing (APOBEC1), restriction of retroviruses (APOBEC3s), and possibly active DNA demethylation [33], [34], [35], [36]. PmCDA1 is involved in the diversification of genes encoding immunoglobulin analogs in sea lamprey and is closely related to other APOBEC enzymes [31]. AID/APOBECs fulfill their functions by catalyzing cytosine deamination, which results in the formation of uracil in the substrate DNA or RNA. Uracil can then be processed by the base-excision repair pathway protein uracil-DNA-glycosylase, followed by repair, which may result in mutations and recombination. If uracil escapes repair during the next round of replication, a C-G to T-A transition occurs (Fig. 2C) [33], [34].

Both HAP treatment (reviewed in [24]) and the ectopic production of PmCDA1 [31], [37] are not very toxic but strongly mutagenic in wild-type yeast as measured by different reporter systems detecting base-pair substitutions. In contrast to the other organisms, HAP does not induce recombination in *Saccharomyces cerevisiae* [24], [38], [39], most likely because a key enzyme required to excise HAP-containing DNA is absent in yeast. In addition, mismatch repair – one of the key safeguards of genome stability [40] – does not seem to recognize HAP in the DNA [24], in contrast to the other base analogs such as dP [41]. These unique properties provide the opportunity to detect a genuine signature of base analog-induced mutations. PmCDA1 was chosen as a prototype of the AID/APOBEC1 family because it has the highest mutagenic effect in the group when produced in yeast [37]. PmCDA1 is recombinogenic in wild-type *S.cerevisiae*, but inactivation of uracil-DNA-glycosylase (*ung1*) completely blocks deaminase-induced recombination [31]. Thus, HAP and PmCDA1 are perfect tools for studying mutagenesis in diploid cells under conditions when induced recombination is suppressed, by mechanism of the induction of independent mutations (Fig. 1, right panel).

We examined the effect of ploidy on HAP- and PmCDA1-induced mutagenesis. The median frequency of canavanine-resistant mutants (Can^R^, mutation in the *CAN1* locus) in the HAP-treated haploid strain LAN201 (see Table S1 for genotypes of strains) is 2.51*10^−5^ (Table 1), a 23-fold increase over the background level. The frequency of HAP-induced Can^R^ mutants in the isogenic diploid strain LAN211 is 5*10^−7^, 833-fold higher than expected based on the mutation frequency in the haploid strain (both copies of the *CAN1* gene have to be inactivated in order to produce an antibiotic resistant phenotype, Fig. 1) (Table 1). We did not find Can^R^ clones in diploids in the absence of mutagen. This was consistent with previous observations that the spontaneous frequency of mutants in wild-type diploids is extremely low [21], [42]. Overall, the results are in full agreement with our earlier genetic data on the mutagenesis of diploids with HAP with a different reporter, the *LYS2* gene (i.e. they are reporter-independent) [21].

**Table 1:** Both HAP and PmCDA1 induce significantly more mutants than expected in diploid strains.

Strain	Ploidy, repair defect	Mutagen	Mutation frequency ×10^−6 ^ a	Fold increase^b^
**Canavanine resistance**				
LAN201	1n	-	1.1 (0.8–1.9)	1
LAN201	1n	HAP	25.1 (21.1–30.2)	23
LAN211	2n	HAP (predicted)	0.0006	0.0006
LAN211	2n	HAP	0.5^c^ (0.4–0.7)	0.46
LAN200	1n, *ung1*	-	7.2 (2.9–9.8)	1
LAN200	1n, *ung1*	PmCDA1	159 (79–254)	22
LAN210	2n, *ung1*	PmCDA1 (predicted)	0.025	0.003
LAN210	2n, *ung1*	PmCDA1	2.3^d^ (1.9–2.7)	0.32
**5-FOA resistance**				
LAN200	1n, *ung1*	-	5.7 (4.1–9.1)	1
LAN200	1n, *ung1*	PmCDA1	264 (116–493)	46
LAN210	2n, *ung1*	PmCDA1 (predicted)	0.07	0.012
LAN210	2n, *ung1*	PmCDA1	2.7^e^ (1.4–3.5)	0.47

a Median and 95% confidence limits.

b With respect to the corresponding haploid.

c 833× over predicted.

d 92× over predicted.

e 39× over predicted.

The expression of PmCDA1 in the *ung1* (uracil-DNA-glycosylase-deficient) haploid strain LAN200 leads to a 22-fold increase in *CAN1* mutagenesis over the background frequency (1.6*10^−4^ *vs.* 7.2*10^−6^). Similar to HAP, the frequency of PmCDA1-induced mutations in the diploid *ung1* strain LAN210 is much higher than expected based on the observed haploid rate (2.3*10^−6^ *vs.* 2.5*10^−8^, a 92-fold increase) (Table 1). Similar results has been obtained with the *URA3* reporter gene (mutants resistant to the 5-FOA). Frequency of FOA^R^ mutants in diploid strain was much higher than predicted based on the measured frequency in the haploid strain (see Table 1). The viability of haploid and diploid cells treated with deaminase was 65% and 90%, respectively.

### Reference genomes

High-throughput “next-generation” DNA sequencing (NGS) has revolutionized biomedical research. In order to better understand the phenomenon of an unexpectedly high mutation rate in diploid strains, we used NGS to determine the genome-wide spectra of mutations induced by HAP and PmCDA1 in yeast. To make the analysis of mutant clones possible, we first determined the sequences of the genomes of our wild-type strains. DNA from LAN201, LAN211, LAN200 and LAN210 (Table S1) was extracted, sequenced on an Illumina HiSeq 2000 instrument, and reference genome sequences were *de novo* assembled from the sequencing data (see *Materials and Methods* for details of sequencing and genome assembly). Since LAN201 and LAN211 — as well as LAN200 and LAN210 — are isogenic to each other, the sequences of their genomes were identical, with the exception of the *MAT* locus. However, the related *UNG1* and *ung1* strains (LAN201 and LAN211 *vs.* LAN200 and LAN210) differ by seven single-nucleotide variations (SNVs), in addition to disruption of the *UNG1* gene by a cassette conferring hygromycin resistance (Table S2). Overall, the sequence of our LAN-specific reference genome contains 12,077,153 bp and covers 92.74% of the S288C nuclear genome. Other genome parameters, such as the number of genes and the GC percent, are similar between the LAN and S288C reference genomes (Table S3).

### Resequencing of HAP-induced haploid and diploid mutants

Next, we resequenced the genomes of canavanine-resistant clones induced by HAP in LAN201 and LAN211 strains. Four haploid and 10 diploid genomes were sequenced. We detected numerous mutations in all 14 genomes (Table 2). All mutations detected in haploid clones have SNV frequencies of 80–100%. This confirms that all cells in the sequenced colony were derived from one mutated progenitor cell. Rare cases where SNV in haploid clones have a frequency between 40 and 80% were assembly errors (see *Materials and Methods*). In diploid clones, most of the mutations are true heterozygous (i.e., frequencies of SNVs between 40 and 80%). Rarely, two or more SNVs in the same gene are found. They could be clustered mutations in one copy of the gene or changes in both copies, i.e. heteroallelic. Such cases in our reporter gene lead to a detectable phenotype due to the inactivation of both copies of the reporter and, therefore, were true heteroalleles. We cannot predict from the sequencing data whether the mutation will be recessive or dominant. However, most of the heterozygous mutations are expected to be recessive, because gain of function is a rather specific event. In addition, not all SNVs lead to the phenotypic changes (also see section “Prediction of effects of multiple SNVs on viability” below). Therefore, it is likely that the functions of the majority of the genes with SNVs are not disrupted in diploid mutants, even in the cases where multiple SNVs were present. In the case of the *CAN1* reporter gene, no dominant mutations have ever been reported, to our knowledge. This is expected because the resistance phenotype is due to the loss of function of Can1p, a one-subunit arginine permease (www.yeastgenome.org). Because of the selection for the loss of function of permease in diploids, two copies of the *CAN1* gene should be damaged (Fig. 1). The predominant mechanism of the appearance of Can^R^ mutants was independent mutations in the two homologs. This results in heteroallelic mutations where both alleles are non-functional as nine clones out of 10 possessed heteroallelic mutations in the *CAN1* gene. One clone had one homozygous mutation and is discussed below.

**Table 2:** Summary of all detected mutations in HAP-treated clones.

Description of group	Strain	Mutation type^a^	T→C	A→G	C→T	G→A	All types	Mutations per 100 Kb^b^
Haploid Can^R^ mutants induced in LAN201	LAN201-1	“heterozygous”	-	-	-	-	-	0.95
		“homozygous”	22	34	33	17	106	
	LAN201-2	“heterozygous”	-	-	-	-	-	0.58
		“homozygous”	19	14	12	20	65	
	LAN201-3	“heterozygous”	-	-	-	-	-	0.48
		“homozygous”	9	10	19	16	54	
	LAN201-4	“heterozygous”	-	-	-	-	-	3.18
		“homozygous”	67	95	85	109	356	
Diploid Can^R^ mutants induced in LAN211	LAN211-1	heterozygous	340	358	164	193	1055	4.75
		homozygous	2	1	2	0	5^c^	
	LAN211-2	heterozygous	301	281	525	504	1611	7.21
		homozygous	0	0	1	1	2	
	LAN211-3	heterozygous	131	115	521	509	1276	5.70
		homozygous	0	0	0	0	0	
	LAN211-4	heterozygous	169	172	620	556	1517	6.83
		homozygous	0	0	5	1	6^c^	
	LAN211-5	heterozygous	117	159	442	537	1255	5.60
		homozygous	0	0	0	0	0	
	LAN211-6	heterozygous	135	123	546	507	1311	5.85
		homozygous	0	0	0	0	0	
	LAN211-7	heterozygous	135	94	680	529	1438	6.43
		homozygous	0	0	1	0	1	
	LAN211-8	heterozygous	122	191	331	628	1272	5.71
		homozygous	1	0	0	2	3	
	LAN211-9	heterozygous	76	137	290	517	1020	4.55
		homozygous	0	0	0	0	0	
	LAN211-10	heterozygous	217	202	652	651	1722	7.91
		homozygous	1	1	11	12	25^c^	
Diploid non-mutants obtained from LAN211 but not selected for Can^R^	LAN211-NM1	heterozygous	39	95	115	225	474	2.23
		homozygous	5	0	8	0	13^c^	
	LAN211-NM2	heterozygous	5	8	12	15	40	0.19
		homozygous	0	0	0	0	0	
	LAN211-NM3	heterozygous	54	49	152	194	449	2.00
		homozygous	0	0	0	0	0	
	LAN211-NM4	heterozygous	22	12	72	55	161	0.72
		homozygous	0	0	0	0	0	
	LAN211-NM5	heterozygous	17	24	127	138	306	1.37
		homozygous	0	0	0	0	0	
	LAN211-NM6	heterozygous	1	1	3	2	7	0.03
		homozygous	0	0	0	0	0	
	LAN211-NM7	heterozygous	146	136	543	441	1266	5.66
		homozygous	0	0	0	1	0	
	LAN211-NM8	heterozygous	21	36	81	94	232	1.04
		homozygous	0	0	0	0	0	

a See Materials and Methods for details of mutation detection. For haploid clones, terms “homozygous” and “heterozygous” are arbitrary and included for simplicity.

b Values for number of mutations are divided by 112.00363 for haploid strains and 224.00726 for diploid strains. Results are rounded to two decimal points. Homozygous mutations in diploids are treated as two heterozygous mutations.

c Majority of homozygous SNVs in diploid clones are results of recombination events (see Table S6 for details). Among five homozygous SNVs found in In LAN211-1, three are grouped together on the distal end of the right arm of ChrV. Among six homozygous SNVs in LAN211-4, four are grouped together on the distal end of left arm of chrV. Among 25 homozygous SNVs in LAN211-10, 22 are found together on the distal end of right arm of chrIV. All 13 homozygous SNVs found in LAN211-NM1 are localized in the left arm of ChrVI. These patches of homozygous mutations found in different clones are not due to the chromosome arm loss, because the coverage of genome assembly for the regions of homozygosity in the corresponding clones is the same as the average coverage throughout the whole genome.

Some mutations in the genomes were found with a SNV frequency of more than 80% and were classified as homozygous. The majority of these rare homozygous mutations in diploid clones apparently result from spontaneous recombination events (see Table 2). This includes the homozygous mutation in the *CAN1* gene of clone LAN211-4, which belongs to the group of 4 homozygous mutations localized on the distal end of the left arm of chromosome V and, therefore, is a result of a mutation-segregation mechanism via recombination (Table 2, Fig. 1). The mutational load is strikingly different in the haploid and diploid clones (*P* <0.005, see *Materials and Methods*). Four haploid clones contain 54 to 356 mutations, whereas diploids had from 1020 to 1747 SNVs per genome (Table 2 and Fig. 2D). The average number of SNVs per 100 Kb is 1.3 for haploids and 6.05 for diploids (Table 2). All mutations are A-T to G-C and G-C to A-T transitions, in agreement with the mechanism of HAP action during replication (Table 2 and Table S4). In most sequenced genomes, mutations in the G-C pairs were more abundant than mutations in the A-T pairs (see right column in Table S4 and Fig. 2D), which is consistent with earlier data with specific reporters [26], [43]. The bias toward mutations in G-C pairs suggests that most of the effects of dHAPTP are attributable to its misincorporation opposite C in the first replication cycle (Fig. 2). However, the variability of the ratio of mutations in the G-C pair to mutations in A-T pairs in individual genomes was high, from 0.5 in LAN211-1 to 5.3 in LAN211-7. In particular, we observed a strong bias toward mutations in A-T pairs in one diploid HAP-induced mutant clone (LAN211-1). The reason for these differences is unknown and may reflect cell-to-cell variability in HAP metabolism and/or DNA replication (see *Discussion*). This highlights the value of whole-genome resequencing studies, which provide a snapshot of the mutagenic process in individual cells.

**Figure 3:**
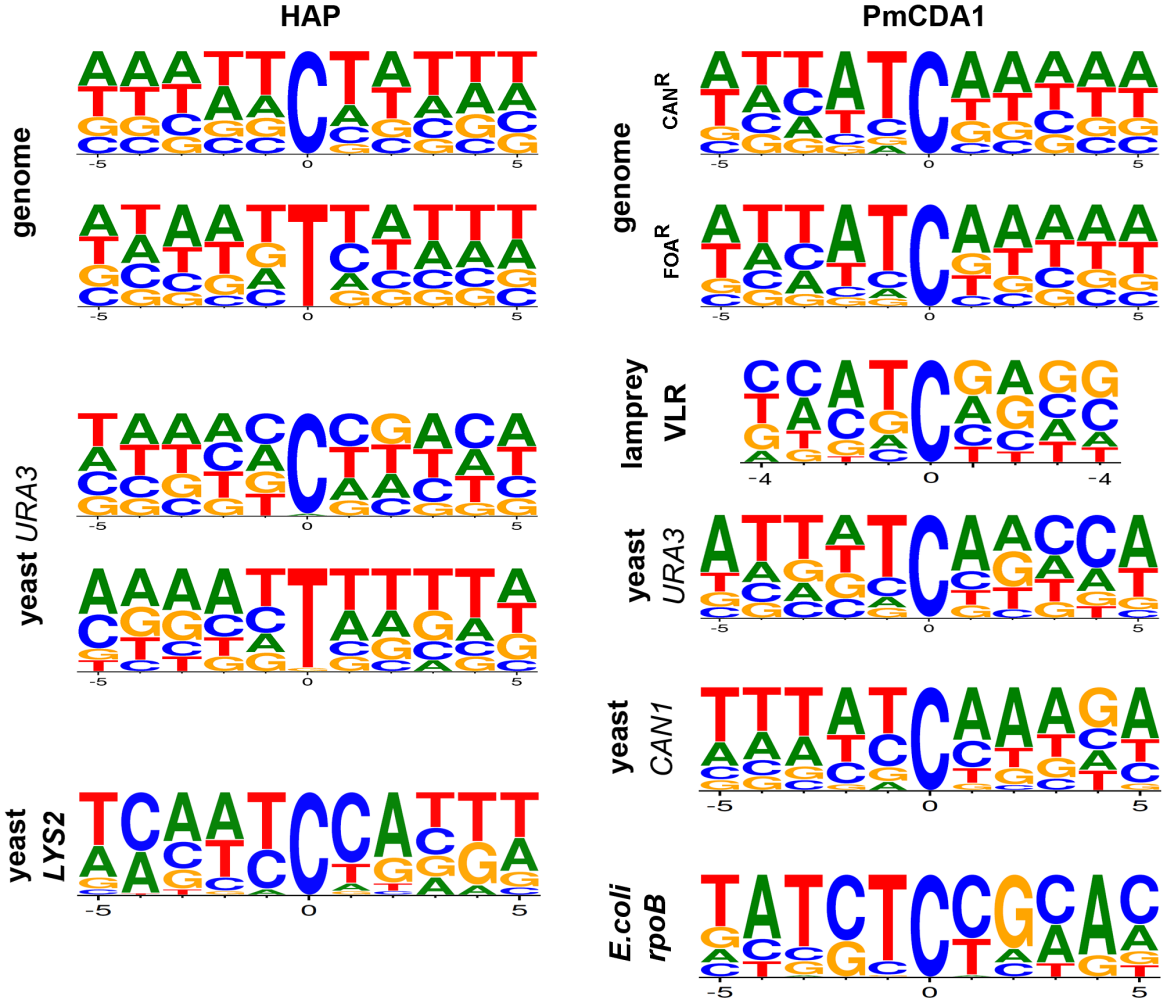
DNA sequence context of HAP- and PmCDA1-induced substitutions. The spectra of mutations induced by HAP in genomes are from this study. Data for the *URA3* reporter is from this work and [26] and for the *LYS2* reporter from [43]. PmCDA1-induced mutation spectra in reporter genes and in lamprey VLRs are a combination of data from this work and [31].

Analysis of the sequence context of these mutations did not reveal any strong biases toward any particular sequence contexts for HAP-induced SNVs (Fig. 3). However, we observed a slight preference for A/T rich sequences in our genome-wide data for both G-C to A-T and A-T to G-C transitions. Mutational spectra obtained using reporter genes shows different results depending on the substitution type and reporter used (Fig. 3, first column of consensus sequences; see *Discussion*).

### Genomes of PmCDA1-induced haploid and diploid mutants

We sequenced four haploid Can^R^, seven diploid Can^R^ and two diploid FOA^R^ (mutations in the *URA3* locus confer resistance to 5-FOA) mutant clones induced by PmCDA1. Similar to the results obtained with HAP, all mutations in haploid PmCDA1-induced mutant strains have SNV frequency >80%, whereas the majority of mutations in diploid clones are heterozygous (Table 3). It is important to note that the average number of mutations in haploids was very close to what was found in yeast *ung1* haploids after induction of hyper-active AID deaminase or APOBEC3B [44]. PmCDA1 induces slightly more homozygous mutants in diploids than HAP. As opposed to the results with HAP-induced diploid mutants (see Table 2), homozygous SNVs in PmCDA1-induced diploid mutants are mostly scattered throughout the genome and, therefore, are not due to recombination events. Even if the homozygous mutations were found very close to each other (such as in the most hypermutable region on chromosome X, see Table S6 and [45] for details), they were always accompanied by the heterozygous SNVs in close proximity, and sometimes the heterozygous SNVs were found in between the homozygous ones. These data indicate that homozygous mutations in genomes of PmCDA1-induced mutants in diploids are unlikely to be due to recombination or gene conversion. It is plausible that regions of the genome that are prone to PmCDA1-dependent deamination can accumulate multiple independent mutations, sometimes leading to the homozygous SNVs. In the *CAN1* reporter, heteroallelic mutations are present in six diploid CAN^R^ mutant clones, while only one mutant clone is homozygous. Both FOA^R^ diploid clones possess heteroallelic SNVs in the *URA3* reporter gene. Diploids accumulate more PmCDA1-induced SNVs than haploids (4.38 *vs.* 0.74 SNVs/100 Kb; 5.9-fold increase; *p* = 0.005); however, the variation of the number of mutations in PmCDA1-induced diploids is higher than in HAP-induced diploid clones (Table 3 and Fig. 2D, 2E). All SNVs are C-G to T-A transitions, as expected from cytosine deamination (Table S5, Table 3, and Fig. 2E). Interestingly, a small fraction of mutations (about 0.6%) are tandem, i.e. two consecutive cytosines or guanines are mutated (CC→TT and GG→AA tandem transitions, see Table S5). We found one triplet CCC→TTT mutation in clone LAN210-FOA-L1. In addition, there are strong regional hot-spots in the genome-wide distribution of PmCDA1-induced mutations that are not present in HAP-induced mutants [45]. The observed local regions which are saturated with mutations cannot be associated with the recombinational hotspots and long regions of ssDNA formed during resection [44], [46], [47] because PmCDA1 does not induce recombination in *ung1* yeast [31]. The high number of hotspots per genome cannot be explained in our system by the spontaneous DSB in yeast cells as it was recently proposed (also see Discussion) [44]. The hotspots of deaminase-induced mutations are described in detail in our recent paper [45] and the underlying mechanisms are currently under investigation.

**Table 3:** Summary of all detected mutations in PmCDA1-treated clones.

Description of group	Strain	Mutation type^a^	C→T	G→A	CC→TT	GG→AA	All types	Mutations per 100 Kb^b^
Haploid Can^R^ mutants induced in LAN200	LAN200-L1	“heterozygous”	-	-	-	-	-	1.25
		“homozygous”	66	73	0	1	140	
	LAN200-L2	“heterozygous”	-	-	-	-	-	0.78
		“homozygous”	57	30	0	0	87	
	LAN200-L3	“heterozygous”	-	-	-	-	-	0.05
		“homozygous”	4	2	0	0	6	
	LAN200-L4	“heterozygous”	-	-	-	-	-	0.89
		“homozygous”	33	67	0	0	100	
Diploid Can^R^ mutants induced in LAN210	LAN210-L1	heterozygous	796	604	7	2	1409	6.47
		homozygous	10	10	0	0	20^c^	
	LAN210-L2	heterozygous	196	241	2	3	442	2.00
		homozygous	1	2	0	0	3^c^	
	LAN210-L3	heterozygous	606	467	4	2	1079	4.87
		homozygous	4	1	0	0	5^c^	
	LAN210-L4	heterozygous	1241	876	10	6	2133	9.71
		homozygous	13	8	0	0	21^c^	
	LAN210-L5	heterozygous	332	296	2	0	630	2.86
		homozygous	2	2	0	0	4^c^	
	LAN210-L6	heterozygous	252	318	2	3	575	2.62
		homozygous	4	2	0	0	6^c^	
	LAN210-L7	heterozygous	251	254	2	0	507	2.34
		homozygous	3	6	0	0	9^c^	
Diploid FOA^R^ mutants induced in LAN210	LAN210-FOA-L1	heterozygous	492	449	3^d^	1	945	4.25
		homozygous	1	3	0	0	4^c^	
	LAN210-FOA-L2	heterozygous	543	546	3	4	1096	4.26
		homozygous	4	3	0	0	7^c^	
Diploid non-mutant clones obtained from LAN210	LAN210-NM1	heterozygous	7	3	0	0	10	0.04
		homozygous	0	0	0	0	0	
	LAN210-NM2	heterozygous	8	6	0	0	14	0.06
		homozygous	0	0	0	0	0	
	LAN210-NM3	heterozygous	2	2	0	0	4	0.02
		homozygous	0	0	0	0	0	
	LAN210-NM4	heterozygous	19	15	0	0	34	0.15
		homozygous	0	0	0	0	0	

a See Materials and Methods for details of mutation detection. For haploid clones, terms “homozygous” and “heterozygous” are arbitrary and included for simplicity.

b Values for number of mutations are divided by 112.00363 for haploid strains and 224.00726 for diploid strains. Results are rounded to two decimal points. Homozygous mutations in diploids are treated as two heterozygous mutations.

c The homozygous mutations were not grouped together (as opposed to the case of HAP) and predominantly localized to the genomic regions enriched for SNVs (see Discussion).

d - one of the SNVs in this clone was triple CCC→TTT substitution.

### Types of HAP-induced mutations near origins of replication

The mutation rate can be affected by the replication timing [48]. The mutagenic mechanism of HAP (Fig. 2) allows for the discrimination of errors on the lagging versus the leading strand during DNA replication [49]. Previous studies examining site-specific reversions reported a preference for HAP-induced errors on the leading strand, when site-specific reversions are studied [49], [50]. Our genome-wide analyses permitted us to reinvestigate this phenomenon independent from the selection for specific mutations. These new, genome-wide analyses of locations of C to T versus G to A mutations found that their distribution is random on the leading or lagging strands. In order to detect potential bias close to the origins of replication, we analyzed mutations around each known origin of replication in the region +/−2000 nucleotides. We extracted all cases of neighboring mutations where two or more mutations are found in the vicinity of the same origin. We assumed that if there is a strand-specific asymmetry of mutations near the origins of replication, this should be reflected by the distribution of the types of neighboring mutations. The changes on the opposite side of the origin of replication should be complementary, because the leading and lagging strands are swapped. For example, if two mutations G-C→A-T are located to the right of an origin, they both should be of the same type, either G→A or C→T, while mutations to the left of this origin should be reciprocal (i.e., C→T and G→A). Analysis of 489 pairs of such neighbor mutations revealed a marginally significant deviation from a random expectation ½: 270 pairs of mutations are consistent with the model of strand-specific asymmetry of mutations, whereas 219 are inconsistent with this model (P sign test = 0.024). This result is in agreement with the model that most errors induced by HAP occur with equal probability on lagging or leading DNA strands, while in some regions/sites the bias could be substantial. Earlier work with site-specific reversions may have only described a minor and specific pathway of HAP mutagenesis at such specific sites [26], [43]. We recently reached the same conclusion for HAP-induced forward mutations in the *URA3* reporter gene [51].

### Genomes of random unselected HAP- and PmCDA1-treated clones (called “non-mutants” in the text)

**Figure 4:**
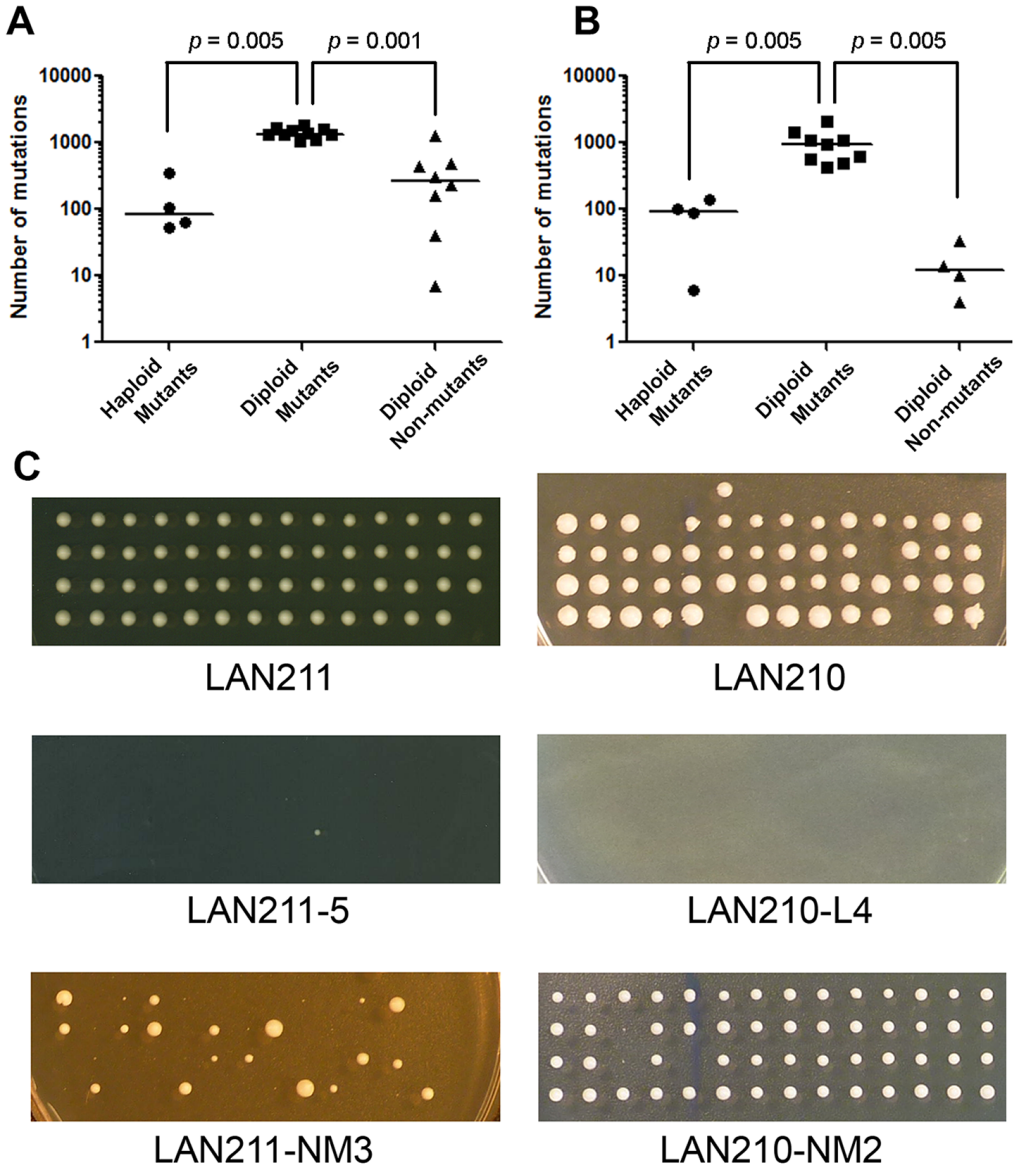
Dependence of mutational load on ploidy and mutant selection. **A.** Number of mutations for different types of HAP-treated genomes. **B.** Number of mutations for different types of PmCDA1-treated genomes. Bars are median values. Note the logarithmic scale. *p* values for Mann-Whitney test are shown for significant differences. **C.** Viability of haploid spores obtained from different diploid clones. Spores resulting from dissecting tetrads produced from wild-type strains (LAN211 and LAN210), mutants induced by HAP (LAN211-5) and PmCDA1 (LAN210-L4), and non-mutant clones treated with HAP (LAN211-NM3) and PmCDA1 (LAN210-NM2) are shown. Each vertical row of four colonies represents the progeny of four haploid spores produced in a single meiosis.

Resequencing of genomes of haploid and diploid HAP- and PmCDA1-induced mutants indicate that there is significant variability in mutation levels in yeast cell populations. Since diploid mutant clones were selected for concomitant mutations of the two copies of the *CAN1* gene, we then investigated the mutational load in cells treated with either mutagen but not selected for canavanine or 5-FOA resistance. We have picked up arbitrary diploid clones from the same YPDU plates that were used to treat strains with HAP before replica-plating to the canavanine-containing media, and from synthetic complete plates that were used to estimate the viability in the case of PmCDA1 (see *Materials and Methods* for details). We sequenced the genomes of eight HAP-treated and four PmCDA1-treated non-mutants. Analysis of SNVs revealed that HAP (Table 2 and Table S4) and PmCDA1 (Table 3 and Table S5) induce the same types of mutations in non-mutant clones as in selected Can^R^ and FOA^R^ mutants, albeit at significantly lower frequencies (Fig. 4A and Fig. 4B, respectively). Most of the mutations in non-mutant clones are heterozygous. Interestingly, HAP-treated non-mutant diploid clones accumulate more SNVs than HAP-induced Can^R^ haploid mutants, whereas PmCDA1-treated non-mutant clones contain fewer SNVs than Can^R^ PmCDA1-induced haploid mutants (Fig. 4). These results provide additiona**l** evidence that levels of HAP- and PmCDA1-induced mutagenesis vary widely, even in the absence of selection (see *Discussion*).

### Viability of haploid progeny of diploid clones with known genome sequence

Recessive heterozygous mutations in diploid genomes have no effect on survival but can cause lethality in haploids. We performed tetrad analysis to estimate the viability of the haploid progeny of wild-type diploid strains, as well as progeny from HAP- and PmCDA1-treated mutant and unselected mutagenized clones. Most of the haploid spores obtained from wild-type strains (LAN211 and LAN210) are viable (Table 4 and Fig. 4C, top row). On the other hand, most of the spores from HAP-induced mutants are inviable (see example in Fig. 4C, second row). A few viable spores were detected for only two mutants tested (LAN211-5 and LAN211-6, see Table 4). Similarly, the majority of spores obtained from PmCDA1-induced mutants do not grow (see e.g. Fig. 4C, second row; see also Table 4). HAP-treated, non-mutant clones show variable viability. All LAN211-NM1 progeny are inviable, whereas viability is very high in LAN211-NM2 and LAN211-NM4 progeny. LAN211-NM3 progeny display an intermediate level of viability (44.4%) and considerable heterogeneity among viable spores. Some of the spores were of normal size, while others were small (Fig. 4C, bottom row; Table 4). The viability of the haploid progeny of PmCDA1-treated non-mutant clones is similar to that of the wild-type strains.

**Table 4:** Viability of haploid progeny of wild-type and mutant yeast strains.

Strain	Number of dissected tetrads	Number of tetrads with viable spores				Survival, %
		4	3	2	1	
LAN211	18	16	2	0	0	97.2
LAN211-1, -2,-3, -4, -7 and -8	72^a^	0	0	0	0	0
LAN211-5	14	0	0	0	3	5.3
LAN211-6	23	0	0	0	1	1.1
LAN211-NM1	28	0	0	0	0	0
LAN211-NM2	26	23	2	2	0	98.1
LAN211-NM3	27	4	22	1	0	44.4^b^
LAN211-NM4	20	19	0	2	0	97.5
LAN210	22	17	5	0	0	94.3
LAN210-L1,-L3, L4	32^a^	0	0	0	0	0
LAN210-L2	12	0	0	0	3	6.25
LAN210-NM1	23	19	3	1	0	94.6
LAN210-NM2	26	25	0	1	0	98.1

a At least 10 tetrads were analyzed for each strain.

b We observed 38 normal-looking colonies. Forty-seven colonies were very small and did not grow any further after transfer to fresh YEPD medium (see Fig. 5).

### Prediction of effects of multiple SNVs on viability

About 75% of HAP-induced mutations were found in open-reading frames (ORFs) of protein-coding genes (Fig. 5A), as expected, given that ORFs encompass about 73% of our reference genomes. Among these mutations, two-thirds (comprising about 50% of all SNVs) are non-synonymous, whereas about one-third (∼25% of all SNVs) are synonymous. SNVs resulting in protein truncations range from 2% to 3% in different genome types (Fig. 5). Interestingly, we found eight mutations predicted to result in the extension of an encoded protein sequence (Table S6). Unexpectedly, we found no difference in the distribution of the types of substitutions between all types of clones - haploid mutants, diploid mutants and diploid non-mutants (Fig. 5).

**Figure 5:**
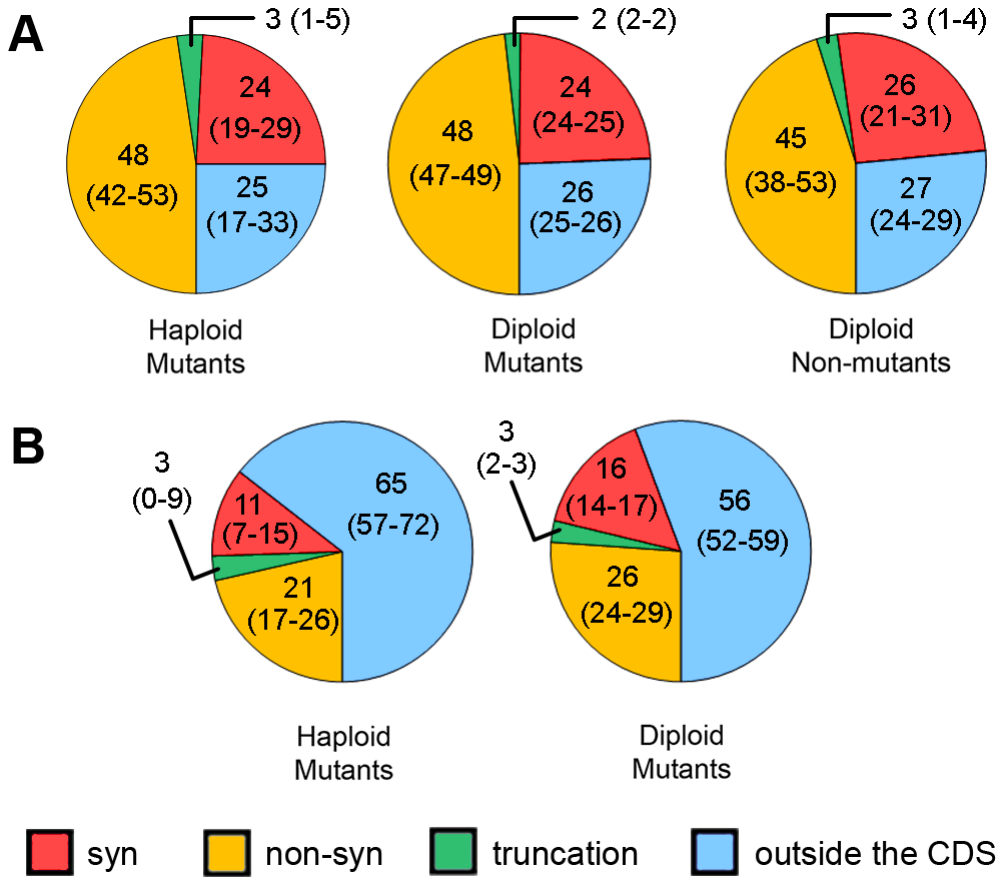
Predicted effects of SNVs on proteins in different types of clones. Results for haploid and diploid mutant and diploid non-mutant clones are shown for HAP (**A**) and for haploid and diploid mutants for PmCDA1 (**B**). Numbers are mean values of percentages with 95% confidence limits in parentheses. PmCDA1-treated, non-mutant clones are excluded from the analysis due to low levels of mutations in their genomes.

The same analysis was performed for SNVs in PmCDA1-induced mutants (Fig. 5B). Here, many more SNVs are present in regions outside of CDS, as compared to the HAP results. Sixty-five and 56 percent of SNVs were found in non-protein coding regions in haploid and diploid mutant clones, respectively. These values are much greater than expected given that non-protein-coding regions comprise only about 25% of the yeast genome. Also, the fraction of non-synonymous SNVs is much less for PmCDA1-induced clones compared to HAP-induced clones (21% and 26% vs. 45–48%). The number of synonymous SNVs for PmCDA1-induced clones ranges from 11% to 16%. The fractions of truncation mutations were similar for HAP and PmCDA1 (3% in PmCDA1 genomes and 2–3% for HAP).

We estimated from our data that 0.3 to 1.4% of all HAP-induced base substitutions cause lethal mutations in haploid cells. Our logic is as follows. Considering that about 18% of yeast genes are essential [52], [53], and given that about half of the SNVs in HAP-treated genomes are either non-synonymous or lead to protein truncation (Fig. 5), we estimate that up to 9% of all SNVs can *potentially* be lethal in haploid progeny. This translates into 43, 4, 40 and 15 such *potentially* lethal SNVs in the genomes of LAN211-NM1, LAN211-NM2, LAN211-NM3 and LAN211-NM4, respectively. To get an estimate of how many of these potentially lethal SNVs are actually lethal, we performed the following calculations. Roughly half (44.4%) of the spores obtained from LAN211-NM3 are inviable, indicating the presence of a single latent lethal heterozygous mutation in this clone. That means that about 2.5% (one mutation out of 40 potentially lethal SNVs) of non-synonymous SNVs in ORFs of essential genes lead to lethality. Strain LAN211-NM1 has a similar number of SNVs but none of its spores are viable (28 tetrads with 112 spores analyzed, all spores inviable; see Table 4). Therefore, spore viability in this strain is less than ∼1% (1/112), which translates into at least six or seven latent lethal heterozygous SNVs in this clone, assuming that the mutations are not linked (viability of spores = (1/2)^n^, where n = number of heterozygous mutations lethal in homozygous state; for 1% viability n≈6.5). At least ∼15% (6.5/43) of the non-synonymous SNVs in essential genes in this clone are lethal. Taken together, our data show that three to 15% of non-synonymous SNVs (or 0.3 to 1.4% of all SNVs) in our HAP-induced mutant clones are lethal in the homozygous state.

## Discussion

### Fraction of HAP or PmCDA1 hypermutable cells

Earlier studies using next-generation sequencing in yeast documented rare spontaneous mutations in yeast haploid and diploid strains [54], [55]. Here, we extend these findings by comparing strains with different ploidy and by applying two different mutagens. We found the intrinsic differences in the ability of cells from the same population to mutate after treatment with two different mutagens. Mutants conferring resistance to canavanine in diploid yeast induced by two types of mutagens accumulate many more SNVs than haploid mutants (Figs. 2D, 2E, 3A, 3B, Tables 2 and 3). This is in agreement with the high mutation frequency observed in diploids (Table 1). The canavanine-resistance phenotype (Can^R^) in diploids is a result of two genetic events needed to inactivate both copies of the *CAN1* gene in diploid strains (Fig. 1). Since both HAP and PmCDA1 (in *ung1* strains) do not induce recombination in our system, both alleles of *CAN1* are inactivated by independent mutations (right branch on Fig. 1), except for rare cases of spontaneous mitotic recombination. Thus, by selection for *can1* mutants in diploid cells, we essentially select the progeny of cells which experienced high levels of mutagenesis.

**Figure 6:**
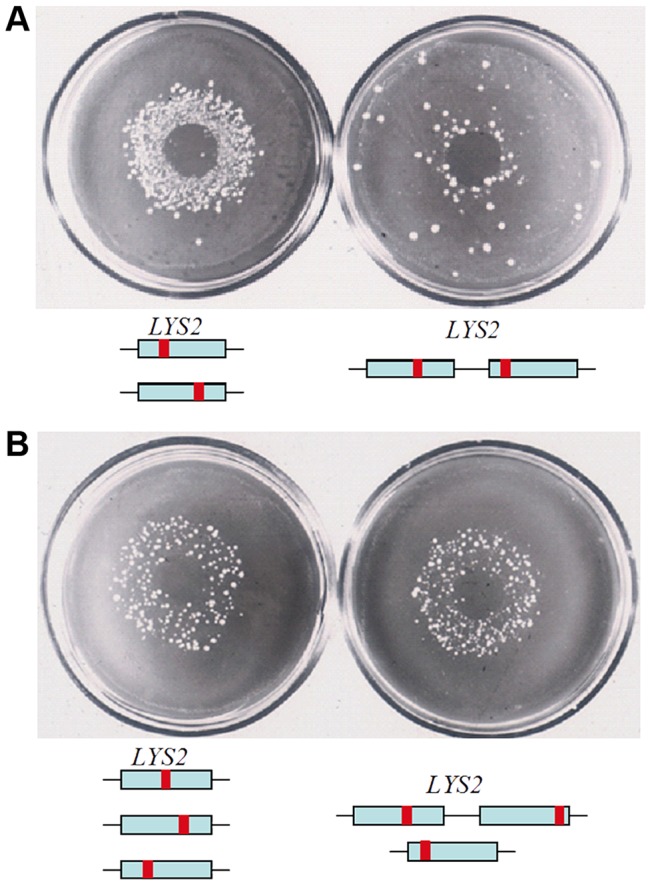
HAP induces mutants in duplicated genes when ploidy is more than one. HAP solution was spotted on a disk in the center of plates and colonies of mutants appear as a circle around the place of application. **A.** Mutant colonies induced with HAP in diploid (left) and a haploid strain with a duplicated *LYS2* reporter (right). **B.** HAP-induced mutants in a triploid strain (left) and diploid strains with duplication of *LYS2* reporter in one homologous chromosome (right).

The effect of transient hypermutability is not specific for Can^R^ selection. First, PmCDA1-induced FOA^R^ diploid mutants possess the same high level of mutations as their Can^R^ counterparts (Fig. 2E, 3B, Table 3). Second, transient hypermutability is observed with other reporters, e.g. using the *LYS2* forward mutagenesis reporter gene [21]. We demonstrated previously that the selection for mutants in haploid strains with a duplication of the reporter gene results in a much smaller number of mutants compared to normal diploids (Fig. 6A) [21] and [56]. The levels of HAP mutagenesis are the same in triploid strains and in diploid strains with a duplicated reporter gene on one of the homologous chromosomes (Fig. 6B). Thus, in these model systems, high levels of mutagenesis require that cells be diploid or have higher ploidy.

**Figure 7:**
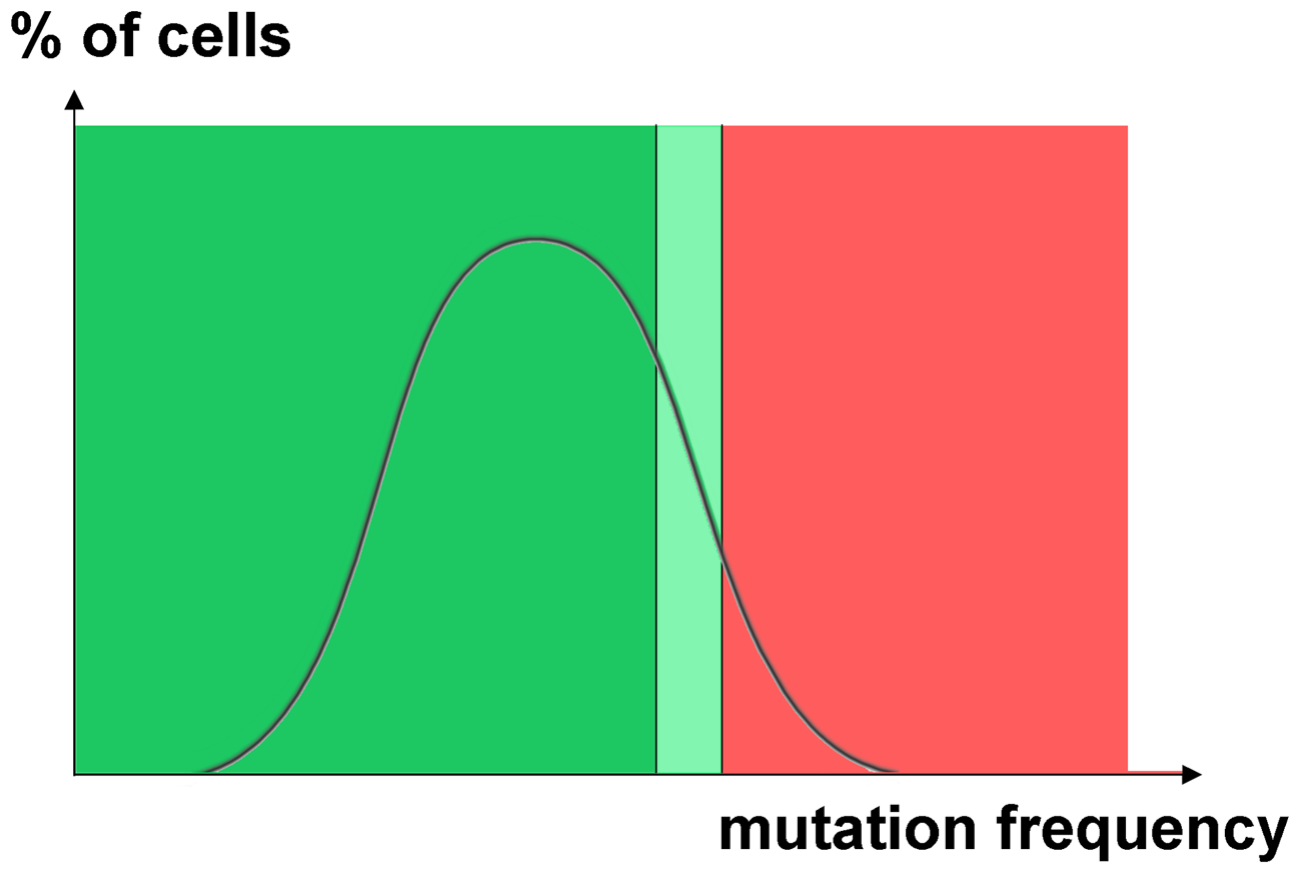
Hypothetical distribution of cells with different induced mutation rates. In the cartoon we used normal distribution as an example. The right side of distribution (highlighted by light green and by red rectangles) contains cells with a very high induced mutation rate. The fraction of hypermutable cells explains the observed increase of mutation frequencies in diploids. The selection of Can^R^ driver mutants in diploids results in the recovery of thousands of passenger mutations. These mutants originated from a transiently hypermutable fraction of cells that survive an extremely high frequency of mutations. Such cells die in haploids (red zone) but survive in diploids. Only the hypermutators from the light green zone survive in haploids. The size of this fraction can be substantial, because mutation avalanches evidence for transient hypermutagenesis was even observed in the genomes of unselected HAP-treated non-mutant clones.

Observed spikes of mutability in individual cells are also not specific to only one particular mutagen. Progeny of such cells was observed in the case of both HAP and PmCDA1, underscoring the fact that different mutagens can induce hypermutagenesis. However, the types of mutations found were mutagen-specific, suggesting that the principal mechanism of mutations in the hypermutable fraction is the same in all other cells. The genome resequencing and genetic results show that the distribution of the mutation load is highly uneven in cell populations. Some cells accumulate dramatically more mutations than others. In other words, the mutation frequency, as virtually any other variable, follows a certain distribution (Fig. 7). Cells that survive very high levels of mutagenesis constitute a hypermutable fraction of a population and impact the overall estimated mutation rate. For example, 1% of cells with a mutation rate three orders of magnitude higher than that in regular cells will elevate the detected rate for a given cell culture by ten-fold. These cells survive in diploid clones and were found as the canavanine-, 5-FOA or aminoadipic acid-resistant mutants that we selected. Haploid cells cannot tolerate such a high level of mutagenesis due to the inactivation of housekeeping genes. The nature and shape of the mutability distribution requires additional investigation with hundreds of genomes from mutagenized but randomly sampled (i.e. non-mutant) clones sequenced.

Since the majority of prior studies on the molecular mechanisms of mutagenesis have been performed in the haploid model systems, the hypermutable fraction of diploid cells described here has evaded detection in the earlier literature. To our knowledge, the only exception is the detection of transiently hypermutable populations of cells that arise during adaptive mutagenesis in bacteria [57], [58], [59], [60]. The existence of these hypermutable bacterial cells is restricted to the specific conditions of nutrient starvation. Importantly, hypermutable cells have never been directly detected in the eukaryotic species, although genetic studies are consistent with their presence [21], [61]. Hypermutable cells can be potentially responsible for the accumulation of multiple mutations during carcinogenesis and evolution.

We further corroborated our model by analyzing the genomes of several non-mutant clones treated with the mutagen. These clones have much less SNVs than their Can^R^ mutant diploid counterparts (Fig. 4). When PmCDA1 is used, the number of SNVs in non-mutant clones is very low (10, 14, 4 and 34 mutations in LAN210-NM1 -LAN210-NM4, respectively), indicating that only a small fraction of cells producing PmCDA1 experience extremely high levels of induced mutagenesis. Therefore, the distribution of cells with different mutation rates is narrower compared to HAP (compare Tables 2 and 3 and Fig. 4A and 3B). It appears that every mutagen causes a different distribution of levels of mutagenesis among cells. The shape of this distribution may be modified by the type of organism, environmental conditions and degree of variation of the mutagen processing physiology in the cells. As a result, the size and parameters of the fraction of hypermutable cells is different for different mutagens. The shape of the “default” distribution of levels of mutagenesis (that is characteristic of a certain cell population not treated with any mutagen) is modified by the application of the mutagen. Mutagens not only increase the integral mutability in the cell population, but they also change the overall shape of the distribution of mutation rates in individual cells as evidenced by the comparison of mutation loads in non-mutant clones treated with HAP (intermediate mutation load) and PmCDA1 (very few mutations).

Several mechanisms could contribute to the uneven mutability of cells in a population. In the case of cells not treated with the mutagens, it could be fluctuations in DNA mismatch repair efficiency in strains with defective DNA polymerase proofreading from cell to cell [22]. In the case of mutagenized cells, the effective intracellular concentration of a mutagen may differ between cells. HAP-induced mutation rates can be influenced by differences in HAP uptake and subsequent metabolism (conversion to dHAPTP by salvage and *de novo* nucleotide synthesis pathways and hydrolysis of dHAPTP by the Ham1 protein [25]). It is known that the deletion of the *HAM1* gene leads to the increase of yeast sensitivity to the mutagenic action of HAP, by almost two orders of magnitude [39]. In the case of PmCDA1, its mutagenesis level could be modulated by differences in deaminase gene expression, protein degradation and aggregation, availability of substrate ssDNA, and fluctuations in levels of proteins that protect the genome from deamination (such as RPA [62]) or stimulate deamination (for example, [63]). The transient hypermutable cells are likely to exist in any cell population. Accumulating evidence suggests that gene expression profiles vary from between cells of the same type in the same tissue (see recent paper about immune cells [64] and references therein). Such single-cell differences may affect the response of the cells to a particular mutagen or induce the expression of mutator proteins, such as APOBEC [9], [10], [14], [16], [42], [44], [45]. However, the mechanisms underlying these effects are different for different organisms, cell types and the mutagen or mutator backgrounds used. The types of mutations found in the progeny of hypermutable cells and their distributions over the genome depends on the conditions, whether cells were mutagenized and, if so, what mutagen was used.

Even when the same mutagen was applied, the level of mutagenesis and its specificity are both variable between different cells. The ratio of mutations in C-G pairs to mutations in A-T pairs varies widely between different HAP-treated clones (Table S4). Moreover, one of the sequenced HAP-induced diploid mutants (LAN211-1) shows a non-typical bias toward A-T to G-C transitions, whereas in other sequenced clones and in published reports using reporter genes, G-C to A-T transitions are more frequent [26], [43]. It is hard to explain this extremely interesting phenomenon of clone-to-clone variability. One possibility is that it could be due to cell-to-cell differences in DNA replication. Eukaryotes replicate DNA with the aid of different polymerases [65]. One can speculate that there is a difference between the main replicative DNA polymerases δ and ε in the rules of HAP incorporation and replication of HAP-containing DNA by these enzymes. In this scenario, if partition between pol δ and ε varies from cell to cell, then this could account for the deviation from the expected behavior during HAP-induced mutagenesis, where more G-C to A-T transitions are typically observed. The use of genome-wide sequencing enabled the detection of both transiently hypermutable diploid cells and cell-to-cell variability in the type of changes induced by the same mutagen in the same population of cells. Similar to new paradigms emerging from single-molecule techniques in biochemistry, our analysis revealed that cells undergoing mutagenesis are not identical and differ significantly from the averaged sample estimates.

### Effects of mutations on viability

Heterozygous mutations in diploid mutants have no effect on fitness as long as they are recessive. To estimate the effects of these mutations on viability, we induced sporulation of diploid yeast clones and dissected the resulting tetrads of haploid spores. The severe decrease in the viability of spores from Can^R^ mutants (Fig. 4C and Table 4) indicates that these diploids possess multiple lethal mutations in the heterozygous state. As expected from their low mutational load, the viability of spores derived from non-mutant PmCDA1-treated diploids is similar to the wild-type level. HAP-treated non-mutant clones show very interesting results after meiosis and tetrad dissection. Although all spores from clone LAN211-NM1 (474 heterozygous SNVs) are inviable, LAN211-NM2 (40 heterozygous SNVs) and LAN211-NM4 (161 heterozygous SNVs) display near-wild-type spore viability. Of the spores from LAN211-NM3, 55.6% (449 heterozygous SNVs) are inviable. Among the LAN211-NM3 clone's spores, 38 formed colonies of normal size and 47 formed very small, barely visible colonies, which were not able to grow any further after being transferred into YPDAU broth and, thus were classified as inviable. Most likely, the ability of haploid spores to grow reflects the segregation of several lethal and conditionally lethal mutations. The segregation pattern differed from one individual spore to another (see Fig. 4C). These results indicate that the upper threshold for the number of heterozygous SNVs per parental diploid genome after mutagenesis that haploid meiotic progeny will tolerate is somewhere around 460.

### The effects of mutations and PmCDA1-induced genome instability

Analyses of the predicted effects of SNVs on genes in different types of HAP-treated clones did not reveal any significant differences in the ratio of synonymous to non-synonymous SNVs and to mutations outside the CDS. PmCDA1-treated clones show similar results, though variability is higher. On the other hand, deaminase induced many more mutations in non-CDS regions than HAP. This result is unexpected because AID/APOBEC deaminases are known to act on ssDNA, especially during transcription [33]. It is possible that PmCDA1 deaminates genomic regions corresponding to 5′- and/or 3′-UTRs of the genes. Another possibility is that deaminases may have access to the ssDNA formed during both transcription and replication in yeast, which results in mutation in transcribed and non-transcribed regions. Further studies are required to clarify the observed effect of preferential enzymatic deamination of non-CDS regions in yeast.

Tandem CC→TT and GG→AA mutations are present in all seven diploid (with one clone possessing triplet CCC→TTT mutation) and one of the haploid PmCDA1-induced mutants. These tandem substitutions are indeed due to enzymatic deamination and not due to the oxidative damage to the DNA, because CC→TT mutations have been found exclusively in the genomes of clones treated with PmCDA1. In addition, dense localized clusters of mutations are present in several loci. These clusters of mutations are highly similar to the clusters recently discovered in yeast under chronic exposure to a mutagen and in human cancers [9], [42]. It has been hypothesized that AID/APOBEC deaminases are involved in the formation of these clusters. The existence of tandem SNVs and mutation clusters induced by PmCDA1 is likely a result of the processive action of deaminase on certain regions of the yeast genome (i.e., where it binds to ssDNA and slides back and forth, catalyzing multiple deaminations) [66], [67]. The processive action of deaminase in the genome may also help to explain the higher numbers of mutations in non-protein-coding regions (Fig. 5). Clones with a high frequency of mutations in ORFs that result from processive deaminase activity are likely to be counter-selected due to the dominant nature of the resulting mutant alleles. Our data provide the direct link between AID/APOBECs and mutational thunderstorms (kataegis); we concentrate on analysis of these clustered mutations induced by deaminase in our parallel paper [45]. Two other groups have recently used a yeast system to study deaminase-induced genome-wide mutagenesis and have come to similar conclusions [44], [68]. Taylor and colleagues [44] proposed (and demonstrated using SceI-induced double-strand break (DSB), see also [47]) that resection of DSB induced by the repair of deaminated cytosine or by other means (independent of deaminase) leads to exposure of ssDNA, which is preferentially deaminated by APOBEC, causing clustered mutations. The Gordenin group proposed similar mechanism [42] and recently reported clustered APOBEC3G-induced mutations in the reporter localized in the overhang resulting from uncapped telomeres [68]. Recombination induced by deaminase is completely blocked by uracil-DNA-glycosylase disruption in our strain [31], but we still observe the genome-wide multiple mutation clusters (this work and [45]). We conclude that the high level of mutagenesis in diploids allowed for the detection of clustered mutations induced independently from recombination. Considering genome-wide distribution on mutations induced by PmCDA1, possible sources of ssDNA for deaminase could be intermediates of replication and transcription.

### Genome-wide analysis of the sequence context of mutations

Whole-genome resequencing provides an unprecedented opportunity to analyze the genome-wide distribution of mutations and their sequence context. We compared the genome-wide mutational sequence context data that we obtained for HAP and PmCDA1 mutagenesis with prior results obtained using reporter genes. We found that HAP has a slight preference for A-T-rich sequences in the genome compared to results obtained using the *URA3* gene as a reporter (Fig. 3, left column of consensus sequences) (data from [26] and this work). An even stronger bias is evident in the spectrum of HAP-induced mutations in the *LYS2* gene, where a major hotspot at position 3165 in the *LYS2* ORF severely affects the results of sequence context analysis (Fig. 3, bottom consensus in the left column) [43]. PmCDA1 mutagenesis shows a strong preference for deamination of cytosines at ATC motifs in the yeast genome, which agrees with the results of the PmCDA1-induced mutational spectra obtained from sea lamprey lymphocyte receptor gene variable regions, further corroborating evidence that PmCDA1 is responsible for VLR diversification [31]. Our genome-wide mutation sequence context results are very similar to the spectra of PmCDA1-induced mutations in the yeast *URA3* and *CAN1* genes when they are used as reporter genes (in this work and [31]). In contrast, the CTC motif (mutated base underlined) is favored by PmCDA1 when the *E.coli rpoB* gene is used as a reporter, which is primarily due to a strong hotspot at position 1592 in the *rpoB* ORF (Fig. 3, bottom consensus, right column) [31]. Taken together, we conclude that analyses of the sequence context preferences of mutagens using reporter genes should be interpreted carefully, especially when the number of detectable positions in the reporter is limited and strong hotspots are found for the reporter/mutagen combination under study.

## Materials and Methods

### Yeast strains

All *S.cerevisiae* strains used in this study (see Table S1 for genotypes) are derived from 1B-D770 [69]. The mutant *ura3–4* allele in this strain was reverted to wild type by transformation with wild type *URA3* DNA obtained by PCR, yielding the LAN201 strain. LAN211 is an auto-diploid of LAN201 obtained by HO endonuclease expression followed by selection for diploids. The haploid *ung1*-deficient strain LAN200 was described previously [62]. Auto-diploidization of LAN200 resulted in the diploid *ung1* strain LAN210.

### Media

Standard yeast media were used [70]. For selection of mutants we have used synthetic complete (SC) agar plates without arginine with 60 mg/L of L-canavanine or 0.1% of FOA. For induction of deaminase expression, minimal synthetic media with addition of 1% raffinose and 2% galactose was used.

### Mutagenesis in yeast

Mutation frequencies were determined by fluctuation analysis as described previously [69]. For the HAP experiment, independent LAN201 or LAN211 clones were grown in rich YPD media overnight. HAP was added to the media, where applicable, to a final concentration 50 µg/ml. After overnight incubation at 30°C, cultures were plated undiluted on synthetic complete media with canavanine (SC+CAN) to select for *can1* mutants, and with dilution to complete (SC) plates to estimate viability. The *CAN1* gene encodes arginine permease, which transports the toxic arginine analog canavanine into cells. Inactivation of *CAN1* renders cells resistant to canavanine.

For the PmCDA1 experiments, plasmid pESC-*LEU*-PmCDA1 was constructed as follows. Total RNA was extracted from the blood of sea lamprey (*Petromyzon marinus*) and reverse-transcribed with oligo (dT). *PmCDA1* was amplified with primers NotICDA1N-F (5′-TTTGCGGCCGCACCATGACCGACGCTGAGTAC, location 118–135 in GenBank accession EF094822) and SpeICDA1C-R (5′- TTTACTAGTGCAACAGCAGGACTCTTAGTG, location 724–742 in EF094822) and cloned into pESC-LEU vector (Stratagene). For yeast experiments, LAN200 or LAN210 strains were transformed with the pESC-LEU-PmCDA1 expression plasmid or with the vector only [31], [37]. Colony-purified transformants were inoculated in 5 ml of synthetic liquid media without leucine containing 1% raffinose. After overnight incubation, galactose was added to cultures at a final concentration of 2%. Galactose activates the GAL1-10 promoter in the pESC-LEU vector which induces the expression of deaminase. After one day of incubation, culture suspensions were plated undiluted on SC+CAN and with dilution on complete plates.

### Isolation of clones for genome sequencing

LAN201 and LAN211 were streaked on YPDAU plates and grown overnight. The next day, they were replica-plated on fresh YPD plates, and a drop of HAP was added to sterile filter paper placed on the agar surface so that different yeast patches receive a similar HAP dose. The next day, streaks were replica-plated on SC+CAN plates to select for mutants. One Can^R^ colony was picked from one streak, then colony-purified and frozen as a glycerol stock at −80°C. To obtain non-mutant HAP-treated clones, yeast from YPD plates with HAP (the same plates used to obtain Can^R^ clones) were streaked on YPDAU plates without HAP and then colony-purified. All of the isolated HAP-treated, non-mutant clones were confirmed to be Can^S^.

In the PmCDA1 experiments, LAN200 and LAN210 were transformed with pESC-LEU-PmCDA1. Individual transformants were then inoculated in 5 ml of liquid synthetic media containing glucose and without leucine, followed by incubation for one day at 30°C with shaking. Cells were then pelleted, washed once with sterile water, then resuspended in 12 ml of synthetic media without leucine containing 2% galactose and 1% raffinose, followed by incubation for 3 days at 30°C with shaking. Aliquots of the resulting yeast suspensions were plated on synthetic complete media containing canavanine to select for *can1* mutants. Aliquots of diluted cultures were plated on synthetic complete (SC) plates to estimate viability. Individual CAN^R^ colonies (one per each independent culture) were colony-purified and stored at −80°C. PmCDA1-treated non-mutant clones were arbitrarily picked up from SC plates. These clones were confirmed to be Can^S^.

### Purification of yeast genomic DNA for sequencing

We used the method described in [71] with slight modifications. Cells were collected from 30 ml of saturated culture (OD_600_≈10) grown in YPDAU medium, washed once with water, and resuspended in 3 ml of lysis buffer (0.1 M Tris-HCl pH 8.0, 50 mM EDTA, 1% SDS). Then 150 µl of 5 M NaCl and ∼1.2 ml of glass beads were added to the suspension. Cells were disrupted by vortexing (2 cycles, 2 min each) in a cold room and then the lysate was centrifuged (13,000 *g*, 10 min). DNA was purified from the supernatant using phenol-chloroform extraction followed by ethanol precipitation. The DNA pellet was dissolved in DNA-grade water and treated with RNAse A (Qiagen, 10 µl of 10 mg/ml per sample, 1 h at 37°C). DNA was purified again by phenol-chloroform extraction followed by ethanol precipitation, and finally resuspended in DNA-grade water. The concentration and quality of DNA preparations were monitored by agarose gel electrophoresis and the use of a NanoDrop spectrophotometer (Thermo Scientific) and a Qubit fluorometer (Invitrogen).

### Library construction and whole-genome resequencing

Isolated yeast genomic DNA was used to construct fragment libraries using the recommended kits for sequencing on the UNMC NGS Core Laboratory's HiSeq 2000 instrument. We multiplexed individual yeast libraries, each derived from an individual clone, in a single lane of an Illumina flow cell. Each of the yeast genomes was sequenced at 100× to 300× coverage (depending on the run) by sequencing 101 bp from each end of the individual DNA fragments in the library (101 bp paired-end sequencing), according to Illumina's recommendations. During the instrument run and after sequencing of the yeast libraries was completed, a variety of quality assurance (QA) measurements were made to ensure the integrity of the DNA sequence data. The DNA “bar codes” used for multiplexing were first used to partition the reads into their respective sample-specific bins, and then the bar codes were stripped from the reads to yield sample-specific DNA sequences of interest. Base-calling error correction was performed on each sample-specific set of de-multiplexed raw reads using Quake [72]. Raw Illumina resequencing data for the LAN211 strain and for the various mutant and non-mutant clones were deposited in the NCBI Sequence Read Archive (www.ncbi.nlm.nih.gov/sra, accession numbers SRA057025 and SRP014741).

### De novo assemblies of reference genomes of parent strains

About ten million pair-end reads generated by sequencing of the whole-genome library obtained from the LAN201 reference strain were used for *de novo* genome assembly using CLC Bio's Genomics Workbench software (CLC Bio, Aarhus, Denmark). This resulted in 458 contigs of various lengths (from 200 to 217,386 bp). These contigs were aligned to the genome of the standard yeast strain S288C using batch BLAST. After sorting of the contigs by chromosome, each set was scaffolded (ordered and oriented) against the corresponding chromosome using Geneious Pro software (Biomatters Ltd, Auckland, New Zealand) [73]. Consensus sequences were extracted from the scaffolds and used in the next step of reference genome assembly. Raw sequencing data (the same 10 million reads that were used for the *de novo* assembly) were then assembled to the extracted consensus sequences, and SNVs were detected using Geneious Pro. After manual identification of false positives and the correction of alignments, a new consensus was obtained. This “version 1” LAN201 draft genome assembly covers 92.74% of the standard S288C yeast genome downloaded from the Saccharomyces Genome Database (SGD, www.yeastgenome.org) in October 2011. This draft assembly has a GC content of 38.24% (compared with 38.31% for S288C; see Table S2). LAN211 is identical to LAN201 except for the mating type locus. To obtain references of *ung1*-deficient strains (LAN200 and LAN210), sequencing reads corresponding to LAN210 were assembled on the LAN211 draft in Geneious Pro. SNVs were called, manually checked, and then a consensus sequence was deduced. This resulted in a LAN210 draft genome assembly. The re-assembly of the LAN200 reads on the LAN210 reference followed by SNV calling confirmed that the two strains are isogenic. These draft genome assemblies were used to analyze the genomes of mutant clones. *UNG1* and *ung1* reference genomes are compared in Table S2.

### Comparative assemblies of genomes of mutant and non-mutant clones treated with the mutagen on reference genomes and SNV calling

Ten to 20 million pair-end reads per clone were comparatively assembled on the LAN211 (for HAP) or LAN210 (for PmCDA1) reference genomes using Geneious Pro. SNVs were called using homozygous (SNV frequency ≥80%) and heterozygous (40% ≤ SNV frequency ≤80%) modes. The threshold SNV call frequencies (40% and 80%) were selected based on pilot experiments designed to optimize the detection of true SNVs and reduce the number of false positives. Regions of high and low coverage (more than two standard deviations from the mean) were excluded from the analysis. At this point, a fraction of non-expected substitution types was observed in the genomes. These included non-G-C→A-T and non-A-T→G-C mutations in HAP-treated genomes and non-G-C to A-T mutations in PmCDA1-treated genomes. The majority of these SNVs were found in the regions where reads were clearly misaligned to the reference (i.e. having low mapping quality). The rest of the non-expected SNVs were found in the otherwise good regions of assembly. We PCR-amplified some of the representative genomic regions where expected and non-expected SNV types were detected, and then sequenced these amplicons using the Sanger method. All SNVs of the expected types were indeed present in the genomes, whereas all non-expected SNVs were found to be assembly errors. For example, we observed frequent putative A→C “transversions” in ACC or ACCCC sequence motifs, but these were not confirmed by Sanger sequencing. Based on these results, we removed all non-standard SNVs from the data set. Finally, detected SNVs were extracted from the alignments for further analyses (Table S6).

### Other bioinformatics techniques

The predicted effects of SNVs on proteins were analyzed in Geneious Pro [73]. To extract the genomic sequence context of mutations, we used *ad hoc* program Rseq1. Consensus sequences, also called sequence logos (Fig. 3) were created using WebLogo 3 (http://weblogo.threeplusone.com/) [74] with adjustment for the GC composition of the corresponding genomes and reporter genes.

### Statistical methods

The Mann-Whitney test as used to compare differences in mutation loads in different types of mutant and non-mutant clones (see Fig. 4). This result suggests that the difference between haploid and diploid strains is significant, reflecting differences in their ability to tolerate the high frequency of induced mutations.

## Supporting Information

Table S1 List of yeast strains used in this work.
(DOC)

Table S2 Nucleotide sequence differences between wild-type and *ung1* reference strains.
(DOCX)

Table S3 Genome assembly parameters of reference strain LAN211 compared to strain S288C from the Saccharomyces Genome Database (www.yeastgenome.org). ^a^ CDS – coding sequence. ^b^ ORF – open reading frame.
(DOCX)

Table S4 Distributions of substitution types (as percentages) in HAP-mutagenized genomes.
(DOCX)

Table S5 Distributions of substitution types (as percentages) in PmCDA1-treated genomes.^ a^ One triple GGG→AAA mutation found.
(DOCX)

Table S6 SNVs detected in sequenced genomes. The table consists of 6 sheets, representing the following data: 1. Mutations detected in the genomes of HAP-induced haploid CANR mutants. 2. Mutations detected in the genomes of HAP-induced diploid CANR mutants.3. Mutations detected in the genomes of HAP-treated diploid non-mutants. 4. Mutations detected in the genomes of PmCDA1-induced haploid CANR mutants. 5. Mutations detected in the genomes of PmCDA1-induced diploid CANR and FOAR mutants. 6. Mutations detected in the genomes of PmCDA1-treated diploid non-mutants. **Description of column names:** Document Name - Name of sequenced clone. Sequence Name - Name of chromosome file. Track Name - Homozygous (80%) or heterozygous (40%) mutation. Minimum - Coordinate of mutation start in reference genome. Maximum - Coordinate of mutation end in reference genome (same as “Minimum” except for tandem mutations).
(XLS)
