# Supplementary material for: Genome-Wide Mutation Avalanches Induced in Diploid Yeast Cells by a Base Analog or an APOBEC Deaminase
Source: PLoS Genet. 2013 Sep 5;9(9):e1003736. doi: 10.1371/journal.pgen.1003736 (PMC3764175; doi:10.1371/journal.pgen.1003736)
Supplement: Table S1 — List of yeast strains used in this work. (DOC) [file pgen.1003736.s001.doc]

**Table S1. List of yeast strains used in this work**

| Strain name | Genotype | Description |
| --- | --- | --- |
| LAN201 | MATa ade5-1 lys2-Tn5-13 trp1-289 his7-2 leu2-3,112 | Haploid Reference. Obtained from 1B-D770 [1] by converting *ura3-4* allele to wild-type by transformation with wt *URA3* PCR product (this work) |
| LAN211 | MATa/MATα ade5-1/ ade5-1 lys2-Tn5-13/ lys2-Tn5-13 trp1-289/ trp1-289 his7-2/ his7-2 leu2-3,112/ leu2-3,112 | Autodiploid of LAN201  Diploid reference (this work) |
| LAN200 | MATa ade5-1 lys2-Tn5-13 trp1-289 his7-2 leu2-3,112 *ung1::hygB* | *ung1* haploid reference. This strain is deficient in uracil-DNA-glycosylase and was obtained from LAN201 by transformation with the *ung1::hygB* PCR product followed by hygromycin selection [2-4]. |
| LAN210 | MATa/MATα ade5-1/ ade5-1 lys2-Tn5-13/ lys2-Tn5-13 trp1-289/ trp1-289 his7-2/ his7-2 leu2-3,112/ leu2-3,112 *ung1::hygB/* *ung1::hygB* | Autodiploid of LAN200  Diploid *ung1-* reference (this work) |

1. Shcherbakova PV, Kunkel TA (1999) Mutator phenotypes conferred by MLH1 overexpression and by heterozygosity for mlh1 mutations. Mol Cell Biol 19: 3177-3183.

2. Lucaccioni A, Pavlov YI, Achilli A, Babudri N (2007) High rate of starvation-associated mutagenesis in Ung(-) yeast caused by the overproduction of human activation-induced deaminase. Curr Genet 52: 239-245.

3. Lada AG, Waisertreiger IS, Grabow CE, Prakash A, Borgstahl GE, et al. (2011) Replication protein A (RPA) hampers the processive action of APOBEC3G cytosine deaminase on single-stranded DNA. PLoS One 6: e24848.

4. Poltoratsky VP, Wilson SH, Kunkel TA, Pavlov YI (2004) Recombinogenic phenotype of human activation-induced cytosine deaminase. J Immunol 172: 4308-4313.
