# Supplementary material for: Genome-Wide Mutation Avalanches Induced in Diploid Yeast Cells by a Base Analog or an APOBEC Deaminase
Source: PLoS Genet. 2013 Sep 5;9(9):e1003736. doi: 10.1371/journal.pgen.1003736 (PMC3764175; doi:10.1371/journal.pgen.1003736)
Supplement: Table S2 — Nucleotide sequence differences between wild-type and ung1 reference strains. (DOCX) [file pgen.1003736.s002.docx]

**Table S2. Nucleotide sequence differences between wild-type and *ung1* reference strains**

| Chromosome | Coordinate | Nucleotide in LAN201 and LAN211 | Nucleotide in LAN200 and LAN210  (*ung1*) | Change | Gene | Codon Change | Protein Effect |
| --- | --- | --- | --- | --- | --- | --- | --- |
| IV | 258870 | G | C | G -> C | - | - | - |
| V | 116512 | A | G | A -> G | *URA3* | GAA -> GAG | None |
| VII | 274887 | C | T | C -> T | *MON1* | GGG -> AGG | Substitution (G -> R) |
| IX | 241678 | A | G | A -> G | *FIS1* | TAT -> TAC | None |
| XIII | Around 230000-231000 | Wild-type *UNG1* gene | *ung1::HygB* | - | *UNG1* | - | Disruption of *UNG1* gene – non-functional uracil-DNA-glycosylase |
| XIV | 96062 | G | A | G -> A | *CUS2* | GTG -> GTA | None |
| XV | 462710 | A | C | A -> C | - | - | - |
| XVI | 693902 | G | A | G -> A | *TKL1* | AGC -> AGT | None |
