# Supplementary material for: Genome-Wide Mutation Avalanches Induced in Diploid Yeast Cells by a Base Analog or an APOBEC Deaminase
Source: PLoS Genet. 2013 Sep 5;9(9):e1003736. doi: 10.1371/journal.pgen.1003736 (PMC3764175; doi:10.1371/journal.pgen.1003736)
Supplement: Table S3 — Genome assembly parameters of reference strain LAN211 compared to strain S288C from the Saccharomyces Genome Database (www.yeastgenome.org). a CDS – coding sequence. b ORF – open reading frame. (DOCX) [file pgen.1003736.s003.docx]

**Table S3. Genome assembly parameters of reference strain LAN211 compared to strain S288C from the Saccharomyces Genome Database (**[**www.yeastgenome.org**](http://www.yeastgenome.org)**)**

| Strain | Genome length  (non-ambiguous nucleotides) | Number of annotated genes | Number of annotated CDS^a^ | %GC | % coding (ORFs) ^b^ |
| --- | --- | --- | --- | --- | --- |
| S288C | 12,077,153 | 6281 | 5870 | 38.31% | 72.46% |
| LAN210 | 11,200,363 (92.74%) | 5928 (94.38%) | 5569 (94.87%) | 38.24% | 73.26% |

^a^ CDS – coding sequence

^b^ ORF – open reading frame
