# Supplementary material for: Genome-Wide Mutation Avalanches Induced in Diploid Yeast Cells by a Base Analog or an APOBEC Deaminase
Source: PLoS Genet. 2013 Sep 5;9(9):e1003736. doi: 10.1371/journal.pgen.1003736 (PMC3764175; doi:10.1371/journal.pgen.1003736)
Supplement: Table S4 — Distributions of substitution types (as percentages) in HAP-mutagenized genomes. (DOCX) [file pgen.1003736.s004.docx]

**Table S4. Distributions of substitution types (as percentages) in HAP-mutagenized genomes**

| Strain | T->C | A->G | C->T | G->A | Mutations in CG pairs / mutations in AT pairs |
| --- | --- | --- | --- | --- | --- |
| Haploid mutants | | | | | |
| LAN201-1 | 20.8 | 32.1 | 31.1 | 16.0 | 0.9 |
| LAN201-2 | 29.2 | 21.5 | 18.5 | 30.8 | 1.0 |
| LAN201-3 | 16.7 | 18.5 | 35.2 | 29.6 | 1.8 |
| LAN201-4 | 18.8 | 26.7 | 23.9 | 30.6 | 1.2 |
| Diploid mutants | | | | | |
| LAN211-1 | 32.3 | 33.9 | 15.7 | 18.2 | 0.5 |
| LAN211-2 | 18.7 | 17.4 | 32.6 | 31.3 | 1.8 |
| LAN211-3 | 10.3 | 9.0 | 40.8 | 39.9 | 4.2 |
| LAN211-4 | 11.1 | 11.3 | 41.0 | 36.6 | 3.5 |
| LAN211-5 | 9.3 | 12.7 | 35.2 | 42.8 | 3.5 |
| LAN211-6 | 10.3 | 9.4 | 41.6 | 38.7 | 4.1 |
| LAN211-7 | 9.4 | 6.5 | 47.3 | 36.8 | 5.3 |
| LAN211-8 | 9.6 | 15.0 | 25.0 | 49.4 | 3.1 |
| LAN211-9 | 7.5 | 13.4 | 28.4 | 50.7 | 3.8 |
| LAN211-10 | 12.5 | 11.6 | 38.0 | 38.0 | 3.2 |
| Diploid non-mutants | | | | | |
| LAN211-NM1 | 9.0 | 19.5 | 25.3 | 46.2 | 2.5 |
| LAN211-NM2 | 12.5 | 20.0 | 30.0 | 37.5 | 2.1 |
| LAN211-NM3 | 12.0 | 10.9 | 33.9 | 43.2 | 3.4 |
| LAN211-NM4 | 13.7 | 7.5 | 44.7 | 34.2 | 3.7 |
| LAN211-NM5 | 5.6 | 7.8 | 41.5 | 45.1 | 6.5 |
| LAN211-NM6 | 14.3 | 14.3 | 42.9 | 28.6 | 2.5 |
| LAN211-NM7 | 11.5 | 10.7 | 42.9 | 34.9 | 3.5 |
| LAN211-NM8 | 9.1 | 15.5 | 34.9 | 40.5 | 3.1 |
