# Supplementary material for: Genome-Wide Mutation Avalanches Induced in Diploid Yeast Cells by a Base Analog or an APOBEC Deaminase
Source: PLoS Genet. 2013 Sep 5;9(9):e1003736. doi: 10.1371/journal.pgen.1003736 (PMC3764175; doi:10.1371/journal.pgen.1003736)
Supplement: Table S5 — Distributions of substitution types (as percentages) in PmCDA1-treated genomes. a One triple GGG→AAA mutation found. (DOCX) [file pgen.1003736.s005.docx]

**Table S5. Distributions of substitution types (as percentages) in PmCDA1-treated genomes**

| Strain | C->T | G->A | CC->TT | GG->AA |
| --- | --- | --- | --- | --- |
| Haploid mutants | | | | |
| LAN200-L1 | 47.1 | 52.1 | 0.0 | 0.7 |
| LAN200-L2 | 65.5 | 34.5 | 0.0 | 0.0 |
| LAN200-L3 | 66.6 | 33.3 | 0.0 | 0.0 |
| LAN200-L4 | 33.0 | 67.0 | 0.0 | 0.0 |
| Diploid mutants | | | | |
| LAN210-L1 | 56.4 | 43.0 | 0.5 | 0.1 |
| LAN210-L2 | 44.2 | 54.7 | 0.4 | 0.7 |
| LAN210-L3 | 56.2 | 43.2 | 0.4 | 0.2 |
| LAN210-L4 | 58.2 | 41.0 | 0.5 | 0.3 |
| LAN210-L5 | 52.8 | 47.2 | 0.3 | 0.0 |
| LAN210-L6 | 44.4 | 55.6 | 0.3 | 0.5 |
| LAN210-L7 | 49.4 | 50.6 | 0.4 | 0.0 |
| LAN210-FOA-L1 | 52.1 | 47.9 | 0.4 | 0.1^a^ |
| LAN210-FOA-L2 | 49.6 | 49.8 | 0.3 | 0.4 |
| Diploid non-mutants | | | | |
| LAN210-NM1 | 70.0 | 30.0 | 0.0 | 0.0 |
| LAN210-NM2 | 57.1 | 42.9 | 0.0 | 0.0 |
| LAN210-NM3 | 50.0 | 50.0 | 0.0 | 0.0 |
| LAN210-NM4 | 55.9 | 44.1 | 0.0 | 0.0 |

^a^ One triple GGG->AAA mutation found.
